# Comparison of Fracture Resistance of Three-Unit Interim Restorations Fabricated by the CAD-CAM Technology With Two Different Milling Machines and the Direct Technique by Using Different Materials

**DOI:** 10.1155/sci5/1608702

**Published:** 2025-05-25

**Authors:** Leyla Sadighpour, Farideh Geramipanah, Mehran Falahchai, Fatemeh Ebrahimi, Hasan Tadbiri

**Affiliations:** ^1^Department of Clinical Sciences, Faculty of Dentistry, University of Toronto, Toronto, Canada; ^2^Department of Prosthodontics, Dental Research Center, Dentistry Research Institute, School of Dentistry, Tehran University of Medical Sciences, Tehran, Iran; ^3^Department of Prosthodontics, Dental Sciences Research Center, School of Dentistry, Guilan University of Medical Sciences, Rasht, Iran

**Keywords:** CAD-CAM, fracture strength, temporary dental prostheses

## Abstract

**Objective:** There is a gap of information regarding the effect of type of milling machine (based on axis number) on mechanical properties of three-unit interim restorations. Thus, this study aimed to compare the fracture resistance (FR) of three-unit interim restorations fabricated by the computer-aided design/computer-aided manufacturing (CAD-CAM) technology with two different milling machines and the direct technique by using different materials.

**Materials and Methods:** Seventy-two three-unit interim restorations were fabricated on mandibular second molar and second premolar metal dies in six groups (*n* = 12) according to the fabrication technique and the material used: milling groups with 4-axis (Arum) and 5-axis (Amann Girrbach) milling machines, and direct fabrication groups by using Visalys, Unifast III, Tempron, and Acropars materials. The restorations underwent thermocycling and cyclic loading, and their FR was measured. Data were analyzed by one-way ANOVA and Tukey post hoc HSD test (*α* = 0.05).

**Results:** Of the directly fabricated restorations, Visalys showed significantly higher FR than other materials (*p* < 0.05) while Acropars showed the lowest FR. The difference in FR between Unifast III and Tempron was not significant (*p*=0.298). The FR of Amann Girrbach and Arum groups was not significantly different (*p*=0.563) but their FR was significantly higher than the FR of other groups (*p* < 0.001).

**Conclusion:** Interim restorations fabricated by CAD/CAM milling had higher FR than traditionally fabricated restorations by the direct technique. Number of axes of the milling machine had no significant effect on FR. Visalys (bis-acrylic material) showed superior results.

## 1. Introduction

Interim restorations are used to protect the prepared teeth in the process of fabrication of final prosthetic restorations. Interim restorations are fabricated aiming to protect the pulp and periodontal tissue against physical, chemical, and thermal damage, preserve the tooth position in dental arch, reinstate the masticatory function, speech, and esthetics, achieve an emergence profile, enable static and dynamic evaluation of the new occlusal scheme, and maintain oral hygiene [1].

Fracture resistance (FR) is an important clinical property of interim restorations, which is defined as resistance against the applied mechanical forces [2]. Fracture is the main cause of failure of interim restorations [2, 3], which can bring about financial and psychological consequences for patients and lead to patient dissatisfaction [2]. In addition to proper design, properties of the restoration materials are also highly important to achieve sufficiently high FR and prevent restoration fracture [3]. Several different materials and methods may be adopted for the fabrication of interim restorations, with different indications and application times [4]. Poly ethyl methacrylates and poly methyl methacrylates (PMMAs) are the most commonly used materials chairside for the fabrication of interim restorations by the direct and indirect techniques [2]. However, bis-acrylic materials are used as the material choice for the conventional fabrication of interim restorations due to their easier application in the oral cavity, optimal color stability, superior mechanical properties, and lower polymerization shrinkage [5].

Interim restorations may be fabricated by different methods, which can be categorized into three groups of direct, indirect, and direct-indirect [1, 6]. The direct (chairside) fabrication technique is commonly adopted for single-crown restorations and is one-step and more cost-effective. Nonetheless, it has drawbacks such as the possibility of tissue damage due to polymerization-generated heat, allergic reaction due to contact of free monomer molecules with the tooth and gingival tissue, lower marginal adaptation and mechanical properties, and lower surface quality due to void formation in the mixing process [7]. Currently, by the advances in technology and introduction of additive and subtractive systems or milling, generally known as the computer-aided design/computer-aided manufacturing (CAD-CAM) technology, they may be used for the fabrication of interim dental restorations [1]. The milling machines can have three, four, five, or more axes. In three-axis machines, the bur moves in three axes of *X*, *Y*, and *Z*. In four-axis machines, in addition to the abovementioned three axes, the material block can also rotate around an additional axis (tension bridge, A). However, in machines with five axes or more, the material block rotates around a higher number of axes [8]. In dentistry, milling machines with three to five axes are more commonly used [9]. The materials used for this purpose include ceramics, polymers, and composite resins that are often composed of polymer materials such as PMMA [6, 7]. In general, the CAD-CAM systems improve the quality, enhance the fabrication process, and decrease the fabrication time of interim restorations [1]. Other advantages of the CAD-CAM systems include high precision, reduction of residual monomers, no temperature rise (due to reaction), and improved marginal adaptation due to absence of polymerization shrinkage, increased density, decreased porosities and residual stress in restoration, easier fabrication process, and optimal clinical function [5, 7].

Several previous studies compared the FR of three-unit interim restorations fabricated by the direct technique and the CAD-CAM systems [3, 6, 10–17], and the majority of them reported higher FR of milled restorations. However, in only a small number of the abovementioned studies, restorations underwent thermomechanical loading for simulation of the clinical setting [3, 15–17]. Moreover, to the best of the authors' knowledge, the effect of type of milling machine (based on the number of axes) on mechanical properties of the fabricated interim restorations has not been previously evaluated. Small number of studies on ceramic restorations revealed that using a five-axis milling machine resulted in fabrication of restorations with higher physical properties [18, 19]. Thus, the purpose of the present study was to compare the FR of three-unit interim restorations fabricated by the CAD-CAM technology with two different milling machines and the direct technique by using different materials. The null hypothesis of the study was that no significant difference would be found in FR of the three-unit interim restorations fabricated by the CAD-CAM technology with two different milling machines and the direct technique by using different materials.

## 2. Materials and Methods

**Figure 1:**
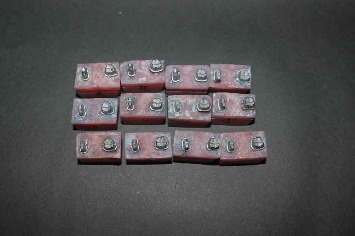
Fabricated models simulating a missing first molar.

In this in vitro study, the sample size was calculated using a formula appropriate for the ANOVA test with the variable “fracture resistance (N).” Based on an effect size of 0.6043, a statistical power of 0.95, and a significance level of 0.05, the minimum sample size required for each group was determined to be 11 [20]. The sample size calculation was performed using the PASS. Seventy-two three-unit interim restorations fabricated by the direct technique by using different materials and also by two different milling machines were evaluated in six groups (*n* = 12): two groups of CAD-CAM milling that were only different in the type of milling machine (5-axis and 4-axis), and four groups fabricated by the direct technique (details mentioned in Table 1). For this purpose, 2 mm of the occlusal surface and 1 mm of the axial walls of a mandibular second premolar and a mandibular second molar with no restoration and caries were removed by a tapered flat-end bur (ISO 856.014, D + Z, Lemgo, Germany) with 4-degree taper. The metal dies were fabricated similar to previous studies [3, 7]. To simulate the periodontal ligament, the root surface of all metal dies was coated with one layer of poly-ether (Impergum F, 3M/ESPE, Seefeld, Germany) with 0.3 mm thickness. The second molar and second premolar metal dies were mounted in autopolymerizing acrylic resin (Technovis 4000; Heraeus Kulzer GmbH & Co, Wehrheim, Germany) to 1 mm below the finish line with 12 mm distance from each other. Accordingly, 72 acrylic blocks, each containing second molar and second premolar metal dies, were obtained (Figure 1).

For indirect (CAD-CAM) fabrication of restorations, Ceramill TEMP PMMA acrylic blocks (light 71 L20nm; Amann Girrbach AG, Koblach, Austria) were used in the Amann Girrbach (AG) group. For this purpose, the mounted metal dies were sprayed by the scan spray (Dentaco GmbH&Co. KG, Germany) and scanned by an optical scanner (Ceramill MaP-400; Amann Girrbach AG, Koblach, Austria). Next, the respective three-unit interim restorations were designed in the CAD software (Ceramill Mind; Amann Girrbach AG, Koblach, Austria). After standardization and confirming the restoration design, PMMA acrylic blocks were milled by a five-axis milling machine (Ceramill, Amann Girrbach AG, Koblach, Austria).

**Figure 2:**
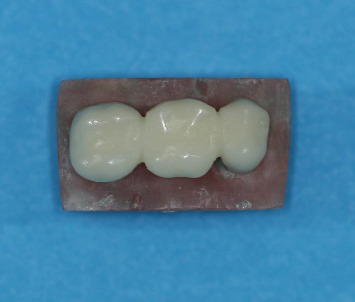
A restoration fabricated by the milling technique on metal dies.

In the second CAD-CAM group (Arum), PMMA acrylic blocks (Ceramill TEMP light 71 L20nm; Amann Girrbach AG, Koblach, Austria) were used similar to the first group (group Ar). Metal dies and full-metal bridges were scanned by an optical scanner (Ceramill MaP-400). All restorations were designed with the same parameters and technique explained for group 1. Finally, PMMA acrylic blocks were milled by a four-axis milling machine (Arum 4X-100; Daewoon, DOOWON ID, Daejeon, Korea) (Figure 2).

For the purpose of standardization of three-unit interim restorations fabricated by the direct technique, a polyvinyl siloxane matrix was obtained from an indirectly fabricated restoration with stops in its peripheral margins. In direct fabrication of restorations, Visalys Temp temporary restorative material was used in group 1 (group Vi), Tempron temporary restorative material was used in group 2, and Acropars (Ac) and Unifast III (Uf) temporary restorative materials were used in groups 3 and 4, respectively. First, a thin layer of petroleum jelly was applied to the metal dies to facilitate removal of the restorations. Each temporary restorative material was then mixed following the manufacturer's instructions. The material was placed into the polyvinyl siloxane index, which was then positioned over the metal dies. A 500 g load was used to fix the index over the dies. The specimens were sequentially polished using silicon carbide (SiC) abrasive papers with progressively finer grits (500, 800, 1200, 2000, and 4000) to achieve a uniform and smooth surface; following polishing, specimens were rinsed with distilled water to remove any debris and air-dried [21]. After polishing, the restorations were stored in water for 24 h. Finally, the interim restorations were inspected under a microscope (Axiolab; Carl Zeiss, Jena, Germany) at × 10 magnification. Restorations with voids were excluded and replaced.

The restorations were thermocycled between 5°C and 55°C with a dwell time of 20 s for a total of 5000 cycles. To simulate oral masticatory forces, the restorations were transferred to a chewing simulator (SD Mechatronik) to undergo 20, 000 cycles of 70 N load with 1.3 Hz frequency applied vertically to the center of the occlusal surface of first molars in distilled water at 25°C for 1 month. Next, all restorations were carefully inspected under a light microscope with a LED source at × 5.8 magnification for detection of initial cracks. Subsequently, the FR of restorations was measured by a universal testing machine (Proline; Zwick/Roell Z010; Zwick/Roell Co., Zwick, Germany) at a crosshead speed of 1 mm/minute. Load was applied by a metal ball measuring 4 mm in diameter to the center of the occlusal surface of first molar in all restorations.

The failure modes for the provisional fixed dental prostheses (FDPs) were classified into the following categories:

  Mesial-distal failure: Fractures involving both the mesial and distal connectors.
  Mesial failure: Fractures localized to the mesial connector.
  Distal failure: Fractures limited to the distal connector.
  Pontic-mesial failure: Fractures originating in the pontic and extending toward the mesial connector.
  Pontic-distal failure: Fractures originating in the pontic and extending toward the distal connector [15].

Data were analyzed by SPSS Version 23 (SPSS Inc., IL, USA). The Shapiro–Wilk test confirmed normal distribution of data, and the Levene test confirmed homogeneity of the variances. Thus, one-way ANOVA was applied to compare the FR, followed by the Tukey HSD post hoc test for pairwise comparisons. Level of statistical significance was set at 0.05.

## 3. Results

No fracture occurred in any restoration during the thermomechanical loading process. According to one-way ANOVA (Table 2), a significant difference existed in FR among different groups (*p* < 0.001). The highest and the lowest mean FRs were recorded in groups AG (806.47 ± 43.75 N) and Ac (392.73 ± 53.44 N), respectively. Pairwise comparisons by the Tukey HSD test (Table 3) revealed no significant difference in FR of restorations fabricated indirectly by the 4-axis and 5-axis milling machines (groups Ar and AG, respectively) (*p*=0.563); however, the FR values in the abovementioned two groups were significantly higher than the FR of other groups (*p* < 0.001). Among the directly fabricated three-unit interim restorations, all differences in pairwise comparisons were significant (*p* < 0.05), except for the difference in FR of Uf and Te groups (*p*=0.298).

The failure mode analysis showed that pontic-related failures (pontic-mesial and pontic-distal) were the most common across all groups. The AG and Arum groups had a higher frequency of pontic failures, with minimal mesial-distal fractures. Similarly, Tempron, Uf, and Visalys groups showed predominance of pontic-mesial failures, while the Ac group exhibited the highest mesial failures. These results highlight the pontic as a critical region prone to stress (Table 4).

## 4. Discussion

The results showed a significant difference in FR of the fabricated three-unit interim restorations. Nonetheless, type of milling machine (according to the number of axes) had no significant effect on mechanical properties. Thus, the null hypothesis of the study was partially rejected.

Of the traditional materials used for the direct technique in the present study, Visalys bis-acrylic material (group Vi) showed the highest FR, and Ac PMMA material (group Ac) showed the lowest FR. Other tested traditional materials had no significant difference with each other in FR, which was in line with previous findings [20, 22, 23]. The observed difference between the bis-acrylic and PMMA materials can be explained from two aspects of molecular structure and preparation process [5]. Higher molecular weight of bis-acrylic monomers compared with methacrylates decreases the mobility and slipping of bis-acrylic polymer fibers and subsequently increases the hardness of the bis-acrylic polymer and reduces the risk of its deformity [20]. Moreover, unlike the methacrylate monomers that only have one active arm and form a polymer with a linear structure, bis-acrylic monomers have two active arms and form polymers with a cross-linked structure and superior physical properties [13]. Superior mechanical properties of autopolymerizing bis-acrylic materials, compared with autopolymerizing PMMA resins, can also be attributed to differences in preparation and the delivery system of the automix cartridge of bis-acrylic resins, compared with the problem of air entrapment and porosities that develop during manual mixing of mono-methyl methacrylates [24]. Additionally, Lawaf et al. [25] demonstrated that Ac had the highest water sorption among different temporary acrylic resin materials, which can explain the lower FR of Ac specimens (group Ac) compared with other traditional materials.

In the present study, the FR of restorations fabricated by the indirect technique (groups Ar and AG) was significantly higher than the FR of traditionally fabricated restorations by the direct technique. The same results were reported in previous studies [3, 5, 10, 13, 14, 17]. In fact, although the linear structure of polymer fibers in mono-methacrylates enhances their water sorption and significantly decreases their strength, prefabricated PMMA blocks by the milling technique are manufactured under high pressure and temperature in the company and do not undergo polymerization shrinkage. Thus, they have a highly cross-linked structure with fewer porosities and small percentage of residual monomers. Thus, they have higher mechanical properties and durability and are not significantly affected by the thermocycling process and mechanical loading [26]. Nonetheless, some previous studies found no significant difference between restorations fabricated directly from di-methacrylates and those milled from mono-methacrylate polymers (indirect technique by using the CAD-CAM technology) [15, 16, 20]. Different materials used by Edelhoff et al. and different fabrication techniques and single-unit design of interim restorations fabricated by Reeponmaha et al. may explain the controversy between their results and the present findings [16, 20]. Sadid-Zadeh et al. [15] used the direct-indirect method for restoration fabrication by the CAD-CAM technology (unlike the use of direct method in the present study). Moreover, the restorations did not undergo thermocycling in their study.

The milling machines vary based on the number of their axes. In their simplest basic design (three-axis), the bur moves along the three axes of *X*, *Y*, and *Z*. The four-axis milling machines are most commonly used in dental offices for the fabrication of inlays, onlays, crowns, and veneers, in which, the bur not only rotates around the aforementioned three axes, the tension bridge containing the material block also rotates around an additional fourth axis [9]. In the five-axis milling machines, in addition to the abovementioned four axes, rotation of the milling spindle also occurs (fifth axis), enabling the fabrication of restorations with a more complex geometry [8]. Beuer et al. [9] in their review study stated that the restoration quality was not affected by the number of milling machine axes. Nonetheless, later in vitro studies in this regard proved otherwise [18, 27]. Alajaji et al. [27] found that the number of axes affected the marginal and internal adaptation of inlay ceramic restorations. Moreover, Bosch et al. [18] demonstrated that five-axis milling machines yielded a higher 3D accuracy than the four-axis milling machines. Thus, number of axes may affect different restoration properties. Therefore, the present study was undertaken to assess the effect of type of milling machine in terms of number of axes on FR of interim restorations for the first time. According to the results, number of axes had no significant effect on FR of interim restorations. However, no previous study is available for the purpose of comparison with the present results.

This study focused on the FR of three-unit interim restorations, providing valuable insights into the performance of CAD-CAM fabricated restorations. However, certain limitations must be acknowledged. The scope of the study was limited to FR, and other mechanical properties, such as flexural strength, surface microhardness, and resistance to dislodgement, were not evaluated. Additionally, marginal sealing, an essential factor for clinical longevity, was not assessed. While the results are specific to the geometry of three-unit FDPs, further studies are needed to evaluate other designs, such as single crowns or larger prostheses. The absence of fractures during fatigue testing highlights the durability of the restorations; however, advanced imaging techniques could provide insights into potential microstructural changes. These considerations pave the way for further research to build upon this study's findings and explore additional aspects of interim restoration performance.

The in vitro design of this study was another limitation. Although the occlusal force concentrated at one point is commonly used in vitro, physiological occlusal loads are rarely applied and concentrated at one point in the oral environment [28]. Therefore, although in vitro studies enable comparison of the performance of materials in a simulated environment, not using cement and inability to precisely simulate the clinical setting in terms of direction and distribution of dynamic occlusal forces may affect interpretation of results and their generalizability to the clinical setting. Similar studies are required on the effect of number of axes of milling machines on other properties of interim restorations. Clinical studies are also recommended to verify the present findings.

## 5. Conclusion

Within the limitations of the present study, it may be concluded that of the traditional materials used for direct fabrication of three-unit interim restorations, bis-acrylic material yielded a higher FR than mono-methacrylate polymers. Moreover, three-unit interim restorations fabricated by different milling machines (four- and five-axis) with the CAD-CAM technology had a significantly higher FR than the directly fabricated restorations. Nonetheless, number of axes of the milling machines had no significant effect on FR of restorations.

## Figures and Tables

**Table 1:** Characteristics of the materials used for the direct technique.

Trade name	Manufacturer	Composition	Mixing time	Working time	Setting time	LT. no	Powder/liquid
Tempron	GC Corporation, Tokyo, Japan	PEMA	20–30 s	1 min	3 min	15-7062	1 gr/0.5 mL
Visalys	Kettenbach GmbH&Co. KG, Germany	Bis-acryl composite (auto)	Auto mix	1 min, 20 s	4 min	141241-67	
Unifast III	GC Dental, Tokyo, Japan	PEMA	10–15 s	1 min	3 min, 10 s	1501292	1 gr/0.5 mL
Acropars	Marlic Co., Tehran, Iran	PMMA	60 s	60 s	3 min	UCB20001-1	1.7 gr/1 mL

**Table 2:** Mean and standard deviation of FR (N) of the study groups (*n* = 12).

Groups	Mean ± SD (*N*)	*p* value (F)
Amman Girrbach	806.43 ± 47.75	< 0.001 (88.80)
Arum	766.63 ± 52.12	
Visalys	632.65 ± 68.42	
Tempron	546.98 ± 65.86	
Unifast III	496.54 ± 61.84	
Acropars	392.73 ± 53.44	

**Table 3:** Pairwise comparisons of the FR of the study groups.

	Amman Girrbach	Arum	Visalys	Tempron	Unifast III	Acropars
Amman Girrbach	—	0.563	< 0.001	< 0.001	< 0.001	< 0.001
Arum		—	< 0.001	< 0.001	< 0.001	< 0.001
Visalys			—	0.008	< 0.001	< 0.001
Tempron				—	0.298	< 0.001
Unifast III					—	0.001
Acropars						—

**Table 4:** Frequency distribution of modes of failure in the study groups.

Failure mode	Groups				
	Mesial-distal	Mesial	Distal	Pontic-mesial	Pontic-distal
Amman Girrbach	0	1	0	7	4
Arum	1	1	0	5	5
Visalys	2	1	0	5	4
Tempron	0	2	1	6	3
Unifast III	0	2	1	6	3
Acropars	0	3	1	6	2

## Data Availability

The datasets used and/or analyzed during the current study are available from the corresponding author on reasonable request. Also, the datasets supporting the conclusions of this article are included within the article.
